# Convalescent plasma transfusion therapy in severe COVID-19 patients- a safety, efficacy and dose response study: A structured summary of a study protocol of a phase II randomized controlled trial

**DOI:** 10.1186/s13063-020-04734-z

**Published:** 2020-10-26

**Authors:** Fazle Rabbi Chowdhury, Ashraful Hoque, Forhad Uddin Hasan Chowdhury, Md. Ruhul Amin, Abdur Rahim, M. Mujibur Rahman, Rubina Yasmin, Md. Robed Amin, Md. Titu Miah, Md. Abul Kalam, Md. Sayedur Rahman

**Affiliations:** 1 Department of Internal Medicine, Bangabandhu Sheikh Mujib Medical University (BSMMU), Dhaka, Bangladesh; 2 Department of Blood Transfusion, Sheikh Hasina National Institute of Burn and Plastic Surgery (SHNIBPS), Dhaka, Bangladesh; 3 Department of Medicine, Dhaka Medical College, Dhaka, Bangladesh; 4 Centre for Medical Biotechnology, Mohakhali, Dhaka, Bangladesh; 5 Kuwait Bangladesh Friendship Government Hospital, Dhaka, Bangladesh; 6 Department of Medicine, Mugda Medical College, Dhaka, Bangladesh; 7 Department of Pharmacology, Bangabandhu Sheikh Mujib Medical University, Dhaka, Bangladesh

**Keywords:** Convalescent plasma, severe COVID-19, Randomised controlled trial, SARS-CoV-2, BSMMU

## Abstract

**Objectives:**

General: To assess the safety, efficacy and dose response of convalescent plasma (CP) transfusion in severe COVID-19 patients

Specific:

a. To identify the appropriate effective dose of CP therapy in severe patients

b. To identify the efficacy of the therapy with their end point based on clinical improvement within seven days of treatment or until discharge whichever is later and in-hospital mortality

c. To assess the clinical improvement after CP transfusion in severe COVID-19 patients

d. To assess the laboratory improvement after CP transfusion in severe COVID-19 patients

**Trial Design:**

This is a multicentre, multi-arm phase II Randomised Controlled Trial.

**Participants:**

Age and sex matched COVID-19 positive (by RT-PCR) severe cases will be enrolled in this trial. Severe case is defined by the World Health Organization (W.H.O) clinical case definition. The inclusion criteria are

1. Respiratory rate > 30 breaths/min; PLUS

2. Severe respiratory distress; or SpO2 ≤ 88% on room air or PaO2/FiO2≤ 300 mm of Hg, PLUS

3. Radiological (X-ray or CT scan) evidence of bilateral lung infiltrate, AND OR

4. Systolic BP < 90 mm of Hg or diastolic BP <60 mm of Hg.

AND/OR

5. Criteria 1 to 4 AND or patient in ventilator support

Patients’ below18 years, pregnant and lactating women, previous history of allergic reaction to plasma, patients who have already received plasma from a different source will be excluded. Patients will be enrolled at Bangabandhu Sheikh Mujib Medical University (BSMMU) hospital, Dhaka medical college hospital (DMCH) and Mugda medical college hospital (MuMCH). Apheretic plasma will be collected at the transfusion medicine department of SHNIBPS hospital, ELISA antibody titre will be done at BSMMU and CMBT and neutralizing antibody titre will be checked in collaboration with the University of Oxford.

Patients who have recovered from COVID-19 will be recruited as donors of CP. The recovery criteria are normality of body temperature for more than 3 days, resolution of respiratory symptoms, two consecutively negative results of sputum SARS-CoV-2 by RT-PCR assay (at least 24 hours apart) 22 to 35 days of post onset period, and neutralizing antibody titre ≥ 1:160.

**Intervention and comparator:**

This RCT consists of three arms, a. standard care, b. standard care and 200 ml CP and c. standard care and 400 ml CP. Patients will receive plasma as a single transfusion. Intervention arms will be compared to the standard care arm.

**Main outcomes:**

The primary outcome will be time to clinical improvement within seven days of treatment or until discharge whichever is later and in-hospital mortality. The secondary outcome would be improvement of laboratory parameters after therapy (neutrophil, lymphocyte ratio, CRP, serum ferritin, SGPT, SGOT, serum creatinine and radiology), length of hospital stay, length of ICU stay, reduction in proportion of deaths, requirement of ventilator and duration of oxygen and ventilator support.

**Randomisation:**

Randomization will be done by someone not associated with the care or assessment of the patients by means of a computer generated random number table using an allocation ratio of 1:1:1.

**Blinding (masking):**

This is an open level study; neither the physician nor the patients will be blinded. However, the primary and secondary outcome (oxygen saturations, PaO2/FiO2, BP, day specific laboratory tests) will be recorded using an objective automated method; the study staff will not be able to influence the recording of these data.

**Number to be randomised (sample size):**

No similar study has been performed previously. Therefore no data are available that could be used to generate a sample size calculation. This phase II study is required to provide some initial data on efficacy and safety that will allow design of a larger study. The trial will recruit 60 participants (20 in each arm).

**Trial Status:**

Protocol version 1.4 dated May 5, 2020 and amended version 1.5, dated June 16, 2020. First case was recruited on May 27, 2020. By August 10, 2020, the trial had recruited one-third (21 out of 60) of the participants. The recruitment is expected to finish by October 31, 2020.

**Trial registration:**

Clinicaltrials.gov ID: NCT04403477. Registered 26 May, 2020

**Full Protocol:**

The full protocol is attached as an additional file, accessible from the Trial’s website (Additional file 1). In the interest in expediting dissemination of this material, the familiar formatting has been eliminated; this letter serves as a summary of the key elements of the full protocol.

## Supplementary information

**Additional file 1.**

## Data Availability

The principal investigator (PI) and an independent two member’s data monitoring committee (DMC) has full access to the data. Data would be made available upon request to the PI during the process of publication if required.

