# Supplementary material for: Convalescent plasma transfusion therapy in severe COVID-19 patients- a safety, efficacy and dose response study: A structured summary of a study protocol of a phase II randomized controlled trial
Source: Trials. 2020 Oct 26;21:883. doi: 10.1186/s13063-020-04734-z (PMC7586693; doi:10.1186/s13063-020-04734-z)
Supplement: Supplementary file 1 — Additional file 1. [file 13063_2020_4734_MOESM1_ESM.pdf]

বঙ্গবন্ধু শেখ মুজিব মেডিক্যাল বিশ্ববিদ্যালয়
Bangabandhu Sheikh Mujib Medical University

শাহবাগ, ঢাকা-১০০০।

Register No:

Received Date:

Meeting Date:

Approved/Not Approved/Revised/Correction

Application for Institutional Review Board (I.R.B) Clearance

1.	Title of the study	:	**Convalescent plasma transfusion therapy in severe COVID-19 patients- a safety, efficacy and dose response phase II RCT**
2.	Principal Investigator (PI)	:	**Prof. Md. Sayedur Rahman**<br>**Chairman, Department of Pharmacology, BSMMU. Mob: 01971840757;**<br>**Email: srkhasru@gmail.com**
3.	Name of present Course	:	N/A
4.	Joining date in Phase-B	:	N/A
5.	Name of Institute	:	N/A
6.	Expected Date of Examination	:	N/A
7.	Guide/Advisor:	:	**N/A**
8.	Co-investigator:	:	**Dr. Fazle Rabbi Chowdhury**<br>Assistant Professor, Department of Internal Medicine, BSMMU<br>Mob: 01916578699; Email: masterfazlerabbi@gmail.com<br><br>**Dr. Md. Ashraful Hoque**<br>Assistant Professor, Department of Transfusion Medicine, Sheikh Hasina Burn and Plastic Surgery Institute<br><br>**Prof. M. Mujibur Rahman**<br>Head, Department of Medicine, Dhaka Medical College<br><br>**Prof. Md. Robed Amin**<br>Professor, Department of Medicine, Dhaka Medical College

Prof. Mohammad Abul Kalam
Professor and Director, Sheikh Hasina Burn and Plastic Surgery Institute (SHBPSI), Dhaka, Bangladesh

Prof. Rubina Yasmin
Head, Department of Medicine, Mugda Medical College

Prof. Md. Titu Miah
Professor of Medicine and Principal, Mugda Medical College

Dr. Sudip Ranjan Deb
Associate Professor, Medicine, Mugda Medical College

Dr. Anisur Rahman
Assistant Professor, Anesthesia, Mugda Medical College

Dr. Tariq Reza
Assistant Professor, Critical Care Medicine, Dhaka Medical College

Dr. Ruhul Amin
Medical Officer, Centre for Medical Biotechnology, Mohakhali

Dr. Abdur Rahim
Junior Consultant (Medicine), Kuwait Bangladesh Friendship Hospital, Dhaka

Dr. Forhad Uddin Hasan Chowdhury
Registrar, Department of Medicine, Medical College

9.	Place of the study	:	BSMMU, DMC, MuMC, Sheikh Hasina Burn & Plastic Surgery Institute, KBFH, CMBT
10.	Type of the study	:	Phase II Randomized Controlled trial
11.	Duration	:	Six (06) months from the date of approval
12.	Total cost	:	15,60, 000/-taka (Fifteen lacs Sixty Thousand Taka only)

13. Funding Agency (If Applicable): : Awaiting confirmation

We agree to obtain approval of the Institutional Review Board of BSMMU for any changes involving the rights and welfare of subjects or any changes of the Methodology before making any such changes.

Principal Investigator   Co-Investigator

Circle the appropriate answer to each of the following

(If not Applicable write NA)

**1. Source of Population :**		
(a) Ill Subjects	Yes	No
(b) Non* Ill Subjects	Yes	No
(c) Minors or persons under guardianship	Yes	No
**2. Does the study involve :**		
(a) Physical risks to the subjects	Yes	No
(b) Social Risks	Yes	No
(c) Psychological risks to subjects	Yes	No
(d) Discomfort to subjects	Yes	No
(e) Invasion of the body	Yes	No
(f) Invasion of Privacy	Yes	No
(g) Disclosure of Information damaging to subject or others	Yes	No
**3. Does the study involve :**		
(a) Use of records, (hospital, medical, death, birth or other)	Yes	No
(b) Use of fetal tissue or abortus	Yes	No
(c) Use of organs or body fluids	Yes	No
**4. Are subjects clearly informed about:**		
(a) Nature and purposes of study	Yes	No
(b) Procedures to be followed including alternatives used	Yes	No
(c) Physical risks	Yes	No
(d) Private questions	Yes	No
(e) Invasion of the Body	Yes	No
(f) Benefits to be derived	Yes	No
(g) Right to refuse to participate or to withdraw from study	Yes	No
(h) Confidential handling of data	Yes	No
(i) Compensation where there are risks or loss of working time or privacy is involved in any particular procedure	Yes	No
**5. Will signed consent form/verbal consent be required :**		
(a) From Subjects	Yes	No
(b) From parent or guardian (if subjects are minors)	Yes	No
**6. Will precautions be taken to protect anonymity of subjects**	Yes	No

TABLE OF CONTENTS

Abstract for IRB 6

CHAPTER ONE: INTRODUCTION

1:1. Introduction 8

1:2. Rationale of the study 9

CHAPTER TWO: MATERIALS AND METHODS

2.1. Research Question 10

2.2. Objectives of the study 10

2:1.1. General objective

2:1.2. Specific objectives

2.3. Study design 10

2.4. Place of study 10

2.5. Study period 10

2.6. Study population 10

2.7. Selection criteria 11

2.7.1. Inclusion criteria

2.7.2. Exclusion criteria

2.8. Endpoint 12

2.9. Randomization 12

2.10. Sample size 12

2.11. Research Instruments 12

2.12. Measures of variables 13

2:13. Procedure of collection of apheretic plasma 13

2.14. Steps of Convalescence plasma transfusion 14

2.15. Procedure of measuring antibody titre 15

2.16. Study flow-chart 16

2.17. Statistical Analysis 17

2.18. Facilities and procedures 17

2.19. Procedure of maintaining confidentiality 18

2.20. Ethical consideration 18

2.21. Quality assurance strategy 19

2.22.	References	19
Annex I:	Budget	21
Annex II:	CRF	22
Annex III:	PIS and Consent for Donor	24
Annex IV:	PIS and Consent for Patient	28

ABSTRACT FOR INSTITUTIONAL REVIEW BOARD (I.R.B)

Project Title: Convalescent plasma therapy in severe COVID-19 patients- a tolerability, efficacy and dose response phase II RCT

Summary: The epidemic of severe acute respiratory syndrome coronavirus 2 (SARS-CoV-2) originating in Wuhan, China, has rapidly spread worldwide[1]. As of March 24, 2020, China had reported 81 767 cases with 3281 deaths, and the World Health Organization (W.H.O) declared coronavirus disease 2019 (COVID-19) a pandemic. As of March 18, 2020, cases were reported in approximately 195 countries [2]. No specific therapeutic agents or vaccines for COVID-19 are available [3]. Several therapies, such as remdesivir and favipiravir, are under investigation,[3,4] but the antiviral efficacy of these drugs is not yet known. The use of convalescent plasma (CP) was recommended as an empirical treatment during outbreaks of Ebola virus in 2014 [5]. A protocol for treatment of Middle East respiratory syndrome coronavirus (MERS-CoV) with CP was established in 2015 [5]. This approach with other viral infections such as SARS-CoV, H5N1 avian influenza, and H1N1 influenza also suggested that transfusion of CP was effective [6-10]. In previous reports, most of the patients received the CP by single transfusion [9-11]. In a study involving patients with pandemic influenza A(H1N1) 2009 virus infection, treatment of severe infection with CP (n = 20 patients) was associated with reduced respiratory tract viral load, serum cytokine response, and mortality [10]. In another study involving 80 patients with SARS, administration of CP was associated with a higher rate of hospital discharge at day 22 from symptom onset compared with patients who did not receive CP [12]. Accordingly, these findings raise the hypothesis that use of CP transfusion could be beneficial in patients infected with SARS-CoV-2. The objective of this study is to describe the initial clinical experience with CP transfusion administered to severe COVID-19 patients. The primary end point of this trial would be to assess the tolerability, efficacy and dose response of CP in severe COVID-19 patients. The secondary end point would be to assess the clinical and laboratory parameters after therapy, in-hospital mortality, length of hospital stay, reduction in proportion of deaths, length of ICU stay, requirement of ventilator and duration of ventilator support. The study will be done at Bangabandhu Sheikh Mujib Medical University (BSMMU), Sheikh Hasina National Institute of Burn & Plastic Surgery (SHNIBPS),

Centre for Medical Biotechnology (CMBT), Dhaka Medical College Hospital (DMCH) and Mugda Medical College Hospital (MMCH). All RT-PCR positive cases with features of severe infection will be enrolled in this study. Patients will be age and sex matched before enrolment since both the variables are important confounder. Apheretic CP will be collected from a recovered patient (consecutive two RT-PCR samples negative) between day 22 to 35 days of recovery and those with the antibody titre above 1:160.

This RCT will consists of three arms, a. standard care, b. standard care and 200 ml CP and c. standard care and 400 ml CP as a single transfusion. Twenty (20) patients will be enrolled for each arm. Randomisation will be done by someone not associated with the care or assessment of the patients by means of a random number table. Allocations will be concealed in sequentially numbered, opaque, sealed envelopes. Clinical parameters [fever, cough, dyspnea, respiratory rate, PaO2/ FiO2 level, pulse, BP, requirement of O2 and others] will be recorded before and after CP. Laboratory parameters such as complete blood count, CRP, chest X-ray, SGPT, SGOT, S. Ferritin and serum antibody titre will be measured before and after transfusion. Allergic or serum sickness like reactions will be noted and adjusted with outcome. Laboratory tests including RT-PCR will be done at BSMMU virology, biochemistry and heamatology department. Apheretic plasma will be collected at the transfusion medicine department of SHNIBPS hospital, ELISA, antibody titre will be done at CMBT, and patients will be enrolled at DMC and MMCH. All necessary screening tests will be done before transfusion.

Graphpad Prism v 7.0 will be used for analysis. Demographic factors and clinical characteristics will be summarized with counts (%) for categorical variables and median (interquartile range [IQR]) for continuous variables, as none is expected to be normally distributed. One way ANOVA test, a non-parametric Mann–Whitney test and a Kruskal–Wallis test will be performed to compare between the arms. For parametric outcomes, we will compare the odds ratios across the pairs.

CHAPTER ONE

1.1: Introduction

To date, there is no specific treatment of proven effect for SARS-CoV infection. The document suggests that current evidence is in favour for testing CP transfusion or other therapeutics, which contain neutralizing antibodies (such as hyper immune immunoglobulin) for treatment of severe SARS-CoV-2 illness (Public Health England 2015).Prior experience in SARS and severe influenza suggest that CP may be considered for patients who are deteriorating (despite other specific and supportive therapy) and in whom the virus remains detectable [12-16].

A recent systematic review of 32 reports from SARS and severe influenza concluded that CP therapy appears safe and may reduce mortality, especially if administered early in the illness [17]. An exploratory post hoc meta-analysis showed a statistically significant reduction in the pooled odds of mortality following treatment compared to placebo or no therapy (odds ratio 0.25; 95 % confidence interval 0.14–0.45; I2 = 0 %)[17]. Citing case series, the authors commented that [1] patients with severe presentations appeared to demonstrate temporal clinical improvements after treatment with CP and [2] administration as early as possible in the diseases course appears to be associated with greatest potential clinical effect.

In another study involving patients with pandemic influenza A (H1N1) 2009 virus infection, treatment of severe infection with CP (n = 20 patients) was associated with reduced respiratory tract viral load, serum cytokine response, and mortality [10]. Study involving 80 patients with SARS, administration of CP was associated with a higher rate of hospital discharge at day 22 from symptom onset compared with patients who did not receive CP [12]. These findings raise the hypothesis that use of CP transfusion could be beneficial in patients infected with SARS-CoV-2.

1.2: Rationale of the Study

Currently no specific treatment is available against COVID-19 infection. Supportive treatment along with concentrated oxygen is the only mean of treatment. Researchers have already opened over 180 clinical trials of potential COVID-19 treatments for recruitment, and nearly 150 could start soon[18]. However, to date, no clinical intervention trial has been completed and reported. Based on anecdotes (small case series), some of the promising drugs could be azithromycin – hydroxychloroquine combination, Lopinavir/ Ritonavir, Tocilizumab, Ramdisivir, Favirinapir and others. CP transfusion is another promising option, which had been tried before in SARS-CoV, MERS-CoV, and Flu and Ebola viral infection. Its effectiveness against COVID-19 yet not explored. Recently published cases series consists of ten cases forecast promising result [19]. Therefore, a phase II RCT to explore the tolerability, efficacy and dose response need to explore urgently.

The cases started to escalate in Bangladesh and the number of confirmed cases already crossed thousand mark. Considering the cost and shortage of other above mentioned anti-viral and biologics, it is inevitable that many of our patients possibly could not afford or get it. However, the CP can be easily collected from the recovered patients and can easily be processed to use it against active infection. This could be an effective and cheap treatment option for a country like ours to face this catastrophe. Therefore conducting a phase II RCT on CP transfusion is the demand of time.

CHAPTER TWO
MATERIALS AND METHODS

2.1 Research question:

Does CP transfusion is tolerable and effective in treating severe COVID-19 patients compared to standard treatment?

2.2. Objectives:

2.2.1. General Objective:

To assess the tolerability, efficacy and dose response of CP transfusion in severe COVID-19 patients

2.2.2. Specific Objectives:

a. To identify the appropriate effective dose of CP therapy

b. To identify the efficacy of the therapy with their end point

c. To examine the clinical improvement after CP transfusion in severe COVID-19 patients

d. To assess the laboratory improvement after CP transfusion in severe COVID-19 patients

2.3. Study design:

Phase II RCT

2.4. Place of study:

Bangabandhu Sheikh Mujib Medical University (BSMMU), Sheikh Hasina Burn & Plastic Surgery Institute, Dhaka Medical College, Mugda Medical College and Centre for Medical Biotechnology

2.5. Study period:

May 2020 – October 2020 (Six months)

2.6 Study population:

COVID=19 positive patients who are diagnosed on the basis of RT-PCR at BSMMU fever clinic or by any other government designated laboratory and admitted at DMCH, MuMC and BSMMU.

2.7. A. Selection Criteria for Patients

2.7.1. Inclusion criteria

Age and sex matched COVID-19 positive (RT-PCR positive) severe cases as per W.H.O and National guideline having the following features

1. Respiratory rate > 30 breaths/min; PLUS
2. Severe respiratory distress; or SpO2 ≤ 88% on room air or $PaO_2/FiO_2 \leq$ 300 mm of Hg, PLUS
3. Radiological evidence of bilateral lung infiltrate, AND OR
4. Systolic BP < 90 mm of Hg or diastolic BP <60 mm of Hg. AND OR
5. Criteria 1 to 4 AND or patient in Ventilator support

2.7.2: Exclusion criteria

1. Patients below18 years
2. Pregnant women and breast-feeding mothers
3. Previous history of allergic reaction to plasma
4. Patient who already received plasma from a different source
5. Those who will not give consent

2.7.B. Donor’s selection criteria

Patients who recovered from COVID-19 will be recruited. The recovery criteria are as follows:

1. Normality of body temperature for more than 3 days
2. Resolution of respiratory tract symptoms
3. Two consecutively negative results of sputum SARS-CoV-2 by RT-PCR assay (at least 24 hours apart).
4. 22 to 35 days of post onset period.
5. Antibody titre ≥ 1:160.

2.8. Clinical End points

2.8.1. Primary Endpoint

The primary outcome will be in-hospital mortality or hospital discharge. This easily measured outcome is the single most important outcome for patients and health care services for COVID-19 infection.

2.8.2. Secondary Endpoints

The secondary endpoint would be improvement of clinical and laboratory parameters after therapy, length of hospital stay, length of ICU stay, reduction in proportion of deaths, requirement of ventilator and duration of ventilator support.

2.9. Randomization and blinding method:

Randomization will be done by someone not associated with the care or assessment of the patients by means of a random number table. In this pilot study, the CP or standard care will not be blinded. However, the primary outcome (oxygen saturations, PaO2/FiO2 and BP) will be recorded using an objective automated method; the study staff will not be able to influence the recording of these data.

2.10: Sample size:

No similar study has been performed previously. Therefore no data are available that could be used to generate a sample size calculation. This pilot study is required to provide some initial data on efficacy and safety that will allow design of a larger study.

2.11: Research instruments

2.11.1. General questionnaire for assessing socio demographic data.

2.11.2. A check list of clinical findings

2.11.3. A check list of investigation findings.

2.12. Measures of Variables

2.12.1. Socio-Demographic variables

- Age
- Gender (Male/Female)
- Living area (Urban/Rural)
- Occupation

2.12.2. Clinical variables:

- Symptoms
- Systolic blood pressure (in mmHg)
- Diastolic blood pressure (in mmHg)
- Temperature
- Respiratory rate
- Heart rate
- GCS
- Proportion of patients developing transfusion reaction

2.12.3. Laboratory variables

- CXR
- CBC
- CRP
- S. Ferritin
- SGPT
- SGOT
- Antibody titre

2.13. Procedure of collection of Apheretic plasma

(a) Pre-donation screening: First of all, 3 ml blood will be drawn from peripheral venous site preferably from dorsum of the hand and will be kept in EDTA tube for further testing. Blood group will be confirmed by using known anti sera. Screening test of 5 diseases (HBs Ag , Anti HCV, HIV, Syphilis and Malaria) will be tested by device method. An automated analyzer will count complete blood count. If all things remains favorable, then next step will be followed.

(b) *Safety assessment:* Body weight and height of the donor will be measured by conventional method (standard digital weight and height scale). For preventing citrate toxicity or hypocalcaemic complications (twitching, numbness, nausea, mild respiratory difficulty etc) 500 mg calcium carbonate will be given to every donor before starting the procedure. Blood pressure and pulse will be monitored in every 10 minute interval for precaution throughout the procedure. Weight, height and CBC parameter will be given in apheresis machine for setting up. Visible and easily palpable anti cubital vein will be selected for venepuncture. Before puncturing the vein, at list 50-60 mm of Hg pressure will be given above the venepuncture area for maintaining sustain pressure. When everything is ready, procedure will be started by puncturing single needle in targeted vein.

2.14. Steps of Convalescent Plasma (CP) transfusion

Before giving CP to the patient blood pressure, pulse, temperature, respiratory rate will be recorded. It can be given through the central venous catheter or by peripheral channel. If peripheral venous site chosen then at least 21g needle should be confirmed. Blood group and cross matching should be confirmed before starting the transfusion. When there is no chance of clerical mistake then CP transfusion can be started at the rate of 100 ml/hour.

Safety Assessment: If patient was suffering from fever then temperature chart should be maintained. When temperature will be raised above 2 degree Fahrenheit from the baseline then transfusion should be stopped and antipyretic should be given according to the body weight. After a pause of 10 minutes transfusion can be started. If patient, feel excessive itching specific area or whole over the body then Injection Chlorpheniramine maleate will be given. Steroid would be preserved for managing severe respiratory distress or uncontrollable itching. Blood pressure, pulse and respiratory will be monitored every 30 minutes interval regularly.

2.15. Procedure of measuring antibody titre

SARS-CoV-2 IgG antibody titer will be tested by ELISA according to the following protocol:

1. At first the serum/plasma samples (10µL) were diluted to 1:40, 1:80, 1:160, 1:320, and 1:640 by serial dilution.
2. 100µl of sample diluents will be added into each well (except blank and control well) which are pre-coated with SARS-CoV-2 antigens.
3. Then 10µl of specimen will be added. 100µl controls will be added into the assigned well directly. All samples will be run in duplicate.
4. The plate will be covered with the plate cover and will be incubated for 30 minutes at 37°C.
5. At the end of the incubation, each well will be washed 5 times with diluted Wash Buffer.
6. 100µl of conjugate will be added into each well except the Blank.
7. Incubation for 20 minutes at 37°C.
8. At the end of the incubation, each well will be washed 5 times with diluted Wash Buffer.
9. 50µl of Substrate Solution A and 50µl of Substrate Solution B will be added into each well. Incubation for 10 minutes at 37°C avoiding light.
10. 50µl of Stop Solution will be added into each well.
11. The plate reader will be calibrated with the Blank well and the absorbance will be read at 450nm.
12. The sample OD value was ≥cutoff OD value will indicate IgG antibody positive.
13. The IgG antibody titre will be determined by endpoint dilution.

Due to lack of COVID-19 plasmid in Bangladesh and global shortage, we will preserve 3 ml serum for each donor and later shipped to peter Medawar building for pathogen research, University of Oxford. Our collaborators in Oxford will conduct the neutralizing antibody titre and we will later match them with each donor. The expenses for neutralizing antibody titre test will be managed by our Oxford colleagues.

2.16: Study flow-chart

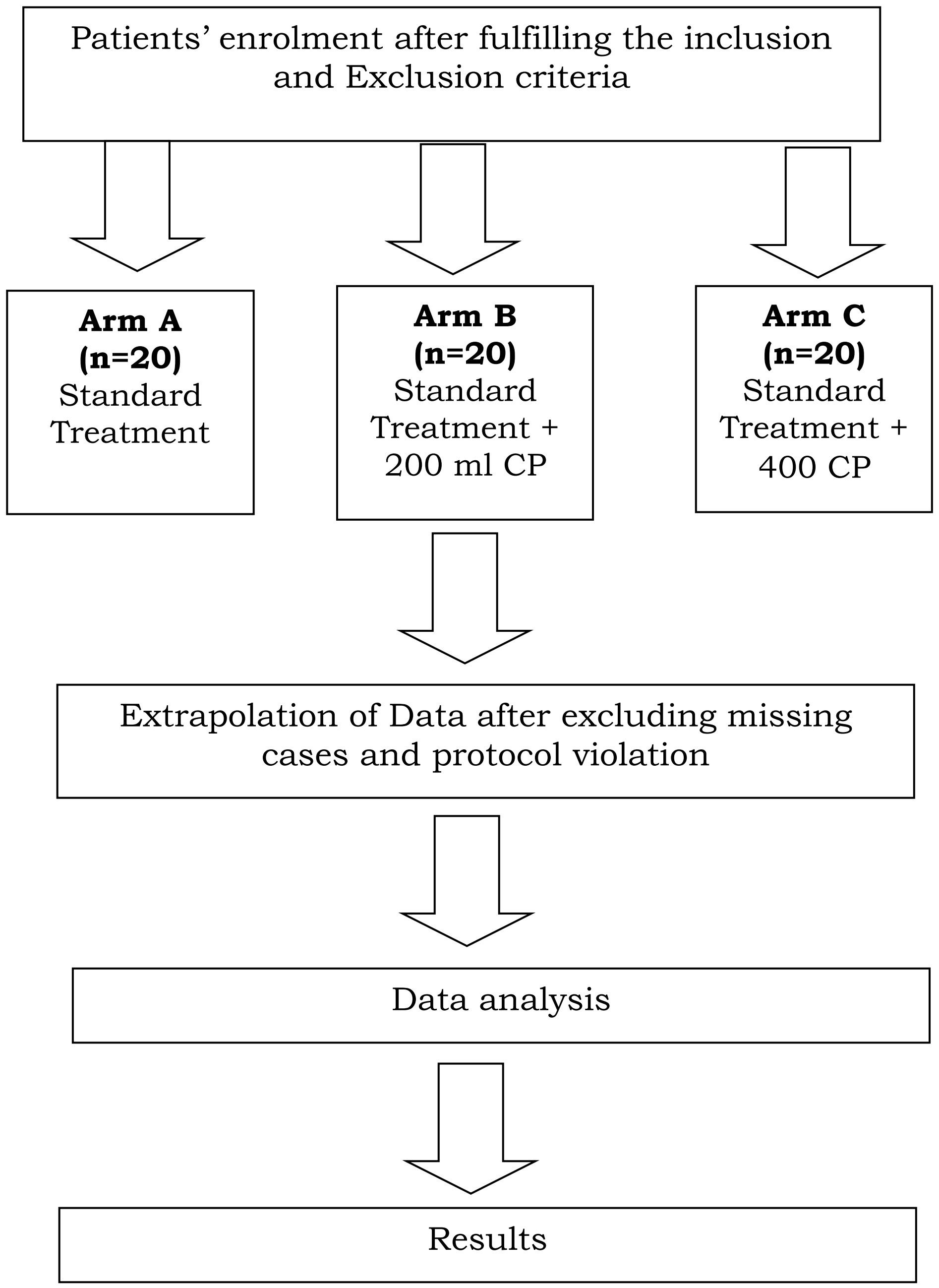

2.17. Statistical analysis:

Graphpad Prism v 7.0 will be used for analysis. Demographic factors and clinical characteristics will be summarized with counts (%) for categorical variables and median (interquartile range [IQR]) for continuous variables. The main analysis will be carried out on an intention-to-treat basis. For the primary outcome, we will compare the mean between three arms using a one-way ANOVA test. We will also do post-hoc analysis in Prism assessing (log-rank Mantel–Cox test) time to sustained saturations >93% and PaO_2/FiO_2<200 mm of Hg censored during enrolment and 3 days after enrolment in a single time point. Relative risk (RR) will be estimated as the number of deaths for person-days until discharge is one of the primary endpoint of the study. For parametric outcomes, we will compare the odds ratios across the pairs (standard arm vs 200 ml CP arm vs 400 ml CP arm). For non-parametric secondary outcomes, all groups will be compared using a Kruskal–Wallis test; if significant, we then planned to perform pairwise comparisons with a non-parametric Mann–Whitney test. For parametric outcomes, we compared the odds ratios across the pairs.

2.18. Facilities and procedure:

The RT-PCR and other study related testing will be done at BSMMU. BSMMU fever clinic already treated more than 1200 COVID-19 positive cases. Potential donors will be tracked from this patient pool. Donors who will agree and give consent will be then send to the Transfusion medicine department of SHBPSI. 5 ml plasma will be collected and sent to the CMBT laboratory for ELISA based antibody titre. If the titre fulfill the desired level (1:160 and above), then 200 ml or 400 ml apheretic plasma (depending on availability and consent of the donor) will be collected on next day. 5 ml serum will be also be collected for sending into the Peter Medawar Building for Pathogen Research, Oxford University for checking the neutralizing antibody titre. This will be send later and matched with the donor. The samples will shipped under the coverage of materials transfer agreement (MTA). Dr. Fazle Rabbi Chowdhury, the Co-PI of this project have necessary permission for shipping samples overseas. In the final stage, the CP will be handover to the clinical team at DMCH and MuMCH as per their demand for transfusion. The clinical team will ensure the

laboratory sample collection before and after CP transfusion and will record the data in the case record form. Department of biochemistry of BSMMU will support the clinical team for lab testing.

2.19. Procedure of maintaining confidentiality:

- Research data will be coded.
- Data will be kept confidential.
- Only the PI and his designated representative will be allowed to access data.
- The soft copy of the data will be kept for 10 years and hard copy for 05 years.
- There is minimum physical, psychological, social risk during collection of blood.
- For blood collection, proper safety method will be followed and samples will be discarded following the lab's standard operative procedure. Serum will be stored to ship in to Oxford. They will store the samples only for neutralizing antibody testing and discard accordingly following local SOP. If the serum is used for another study, additional permission will be sort from the ethics committee.
- For safeguarding confidentiality and protecting anonymity each of the patient will be given a special ID no.
- A signed informed consent will be taken from the patient convincing that privacy of the patient will be maintained.

2.20. Ethical considerations:

All the participants will be informed in detail about the nature and purpose of the study. Before obtaining the consent all patient and or their attendant (must have to be first degree relative who can legally represent the patient) will be explained about the detail study procedure, the benefits & potential risk of (if any) involved with the study. Informed and understood written consent will be taken from every patient or from his or her attendant before enrollment. Each & every patient will enjoy right to participate, refuse and withdraw from the study at any point of the study. Patient enrollment will only started after obtaining ethical approval by the "Institutional Review Board" of BSMMU. The study will not involve any additional investigation that might cause financial burden to the patients. Every patient has freedom to quit. Confidentiality anonymity will be maintained. Data collected from the patient will be considered as confidential and kept by principal investigator under adequate security and restricted accessibility for purposeful use only. Patients will be

provided and ID number & contact number of investigator & guide so that they will be able to contact for any query regarding the study & their health condition, treatment & laboratory reports.

2.21. Quality assurance strategy:

Patient selection criteria & ethical issue will be strictly maintained during patient enrollment for the study. An independent two member's data monitoring committee (DMC) will be formed. The committee will check the data at least twice during the enrollment period. The Co-PIs will inform any serious adverse events (SAE) and suspected unexpected serious adverse reaction (SUSAR) to PI within 6 hours of incidence and the PI will inform this to DMC and Ethical review board by 24 hours. If any change or correction of questionnaire and in data collection strategy is required, it will be done as per advice of the DMC. PI to check the consistency of data collection will do midterm evaluation of the study. The PI and his team can share the interim result to media before formal publication considering the importance of this trial. Data cleaning will be done before editing in the computer for analysis.

2.22. References:

1. Huang C, Wang Y, Li X, et al. Clinical features of patients infected with 2019 novel coronavirus in Wuhan, China. Lancet. 2020;395(10223):497-506.
2. WHO. Novel coronavirus (COVID-19) situation. Updated March 24, 2020. https://experience. arcgis.com/experience/ 685d0ace521648f8a5beeeee1b9125cd
3. Wu Z, McGoogan JM. Characteristics of and important lessons from the coronavirus disease 2019 (COVID-19) outbreak in China: summary of a report of 72 314 cases from the Chinese Center for Disease Control and Prevention. JAMA. 2020. Published online February 24, 2020. doi:10.1001/ jama.2020.2648
4. Lu H. Drug treatment options for the2019-newcoronavirus (2019-nCoV). Biosci Trends. 2020;14(1):69-71.
5. Chen L, Xiong J, Bao L, Shi Y. Convalescent plasma as a potential therapy for COVID-19. Lancet Infect Dis. 2020;S1473-3099(20)30141-9.
6. Kraft CS, Hewlett AL, Koepsell S, et al; Nebraska Biocontainment Unit and the Emory Serious Communicable Diseases Unit. The use of TKM-100802 and convalescent plasma in 2 patients with Ebola virus disease in the United States. Clin Infect Dis. 2015;61(4):496-502.
7. van Griensven J, Edwards T, de Lamballerie X, et al; Ebola-Tx Consortium. Evaluation of convalescent plasma for Ebola virus disease in Guinea. N Engl J

Med. 2016;374(1):33-42.

8. Florescu DF, Kalil AC, Hewlett AL, et al. Administration of brincidofovir and convalescent plasma in a patient with Ebola virus disease. Clin Infect Dis. 2015;61(6):969-973.

9. Zhou B, Zhong N, Guan Y. Treatment with convalescent plasma for influenza A (H5N1) infection. N Engl J Med. 2007;357(14):1450-1451.

10. Hung IF, To KK, Lee CK, et al. Convalescent plasma treatment reduced mortality in patients with severe pandemic influenza A (H1N1) 2009 virus infection. Clin Infect Dis. 2011;52(4):447-456.

11. Burnouf T, Radosevich M. Treatment of severe acute respiratory syndrome with convalescent plasma. Hong Kong Med J. 2003;9(4):309.

12. Cheng Y, Wong R, Soo YO, et al. Use of convalescent plasma therapy in SARS patients in Hong Kong. Eur J Clin Microbiol Infect Dis. 2005;24 (1):44-46.

13. Yang Y, Yang M, Shen C, et al Evaluating the accuracy of different respiratory specimens in the laboratory diagnosis and monitoring the viral shedding of 2019-nCoV infections. Preprint. medRxiv. Preprint posted online February 17, 2020. doi:10.1101/2020.02.11.20021493

14. Villar J, Blanco J, del Campo R, et al; Spanish Initiative for Epidemiology, Stratification & Therapies for ARDS (SIESTA) Network. Assessment of PaO2/FiO2 for stratification of patients with moderate and severe acute respiratory distress syndrome. BMJ Open. 2015;5(3):e006812. doi:10. 1136/bmjopen-2014-006812

15. Liu C, Yang Y, Gao Y, et al Viral architecture of SARS-CoV-2 with post-fusion spike revealed by Cryo-EM. bioRxiv. Preprint posted online March 5, 2020. doi:10.1101/2020.03.02.972927

16. Yeh KM, Chiueh TS, Siu LK, et al. Experience of using convalescent plasma for severe acute respiratory syndrome among healthcare workers in a Taiwan hospital.J Antimicrob Chemother. 2005; 56(5):919-922.

17. Mair-Jenkins J, Saavedra-Campos M, Baillie JK, et al; Convalescent Plasma Study Group. The effectiveness of convalescent plasma and hyper-immune immunoglobulin for the treatment of severe acute respiratory infections of viral etiology: a systematic review and exploratory meta-analysis. J Infect Dis. 2015;211(1):80-90.

18. Zhu R, Gao R, Robert S, Gao J, Yang S, Zhu C. Systematic Review of the Registered Clinical Trials of Coronavirus Disease 2019 (COVID-19). medRxiv doi: https://doi.org/10.1101/2020.03.01.20029611.

19. Duana K, Liuc B, Lid C, Zhange H , Yuf T, Qug J, et al. Effectiveness of convalescent plasma therapy in severe COVID-19 patients. Proc Natl Acad Sci U S A. 2020 Apr 6. pii: 202004168. doi: 10.1073/pnas.2004168117.

Case No: A/B/C-

Annex-I: CRF

Title: Convalescent plasma transfusion therapy in severe COVID-19 patients- a tolerability, safety and dose response Phase II RCT

**Study Arm: A (Standard)/ B (Stand+200ml CP)/ C (Stand+400ml CP)**<br>**Date of Entry:**					
**Patient code number/ Hospital registration number**					
Name (name will not be used in public domain):					
Age (years or mo)(:			Sex: Male /Female		
Address					
Division			District		
Upazilla/Thana			Union		
Ward			Road		
House			National ID if known:		
Occupation			Contact No:		
Date of symptom onset .…./…../2020					
Date of sample positive ……/…../2020					
Date of admission: ……/……/2020					
**1. General Symptoms (Please tick if present):**	Yes =1	No= 2		Yes =1	No= 2
Fever			Fatigue		
Cough			Anorexia		
Dyspnoea			Headache		
Altered sense of smell			Confusion		
Altered sense of taste			Nasal congestion		
Sore throat			Conjunctivitis		
Dairrhoea			Dizziness		
Vomiting			Chest pain		
Myalgia			Others (Mention)		
**2.Signs:**					
Cyanosis			Admission SpO2		%
Tachypnoea			GCS		15
Crackles or bronchial breath sound on auscultation Yes/No					
**3.Vital signs during admission:**					
Body temperature (axillary)…………… ^{o}F			Heart rate………./min		
Blood pressure ………/………. mmHg			Respiratory rate……./min		

**4. Co-morbidities/ Risk factors**		
DM: Yes/No	CLD: Yes/No	Chr Heart Disease: Yes/No
HTN: Yes/No	Smoking: Yes/No	CVD: Yes/No
Asthma: Yes/No	Recent Surgery: Yes/No	COPD: Yes/No
CKD: Yes/No	Recent Chemotherapy: Yes/No	Malignancy: Yes/No
Chronic Liver Disease	Obesity: Yes/No	HIV: Yes/ No

Case No: A/B/C-

Annex-I: CRF

Title: Convalescent plasma transfusion therapy in severe COVID-19 patients- a tolerability, safety and dose response Phase II RCT

**Study Arm: A (Standard)/ B (Stand+200ml CP)/ C (Stand+400ml CP)**<br>Date of Entry:					
**Patient code number/ Hospital registration number**					
Name (name will not be used in public domain):					
Age (years or mo)(:			Sex: Male /Female		
Address					
Division			District		
Upazilla/Thana			Union		
Ward			Road		
House			National ID if known:		
Occupation			Contact No:		
Date of symptom onset .…./…../2020					
Date of sample positive ……/…../2020					
Date of admission: ……/……/2020					
**1.General Symptoms (Please tick if present):**	Yes =1	No= 2		Yes= 1	No=2
Fever			Fatigue		
Cough			Anorexia		
Dyspnoea			Headache		
Altered sense of smell			Confusion		
Altered sense of taste			Nasal congestion		
Sore throat			Conjunctivitis		
Dairrhoea			Dizziness		
Vomiting			Chest pain		
Myalgia			Others (Mention)		
**2.Signs:**					
Cyanosis			Admission SpO2		%
Tachypnoea			GCS		15
Crackles or bronchial breath sound on auscultation<br>Yes/No					
**3.Vital signs during admission:**					
Body temperature (axillary)…………… °F			Heart rate………./min		
Blood pressure ………/………. mmHg			Respiratory rate……./min		

**4. Co-morbidities/ Risk factors**		
DM: Yes/No	CLD: Yes/No	Chr Heart Disease: Yes/No
HTN: Yes/No	Smoking: Yes/No	CVD: Yes/No
Asthma: Yes/No	Recent Surgery: Yes/No	COPD: Yes/No
CKD: Yes/No	Recent Chemotherapy: Yes/No	Malignancy: Yes/No
Chronic Liver Disease	Obesity: Yes/No	HIV: Yes/ No

5. Laboratory Profile (Fill up the blanks)

	Baseline	24 hours after CPT	72 hours after CPT	7 days hours after CPT
**Serum antibody titre**			x	x
**CBC**	TC:<br>WBC:<br>N: L:<br>PCV: Hb:<br>ESR:	x		x
**CRP**		x		x
**SpO_2 (before transfusion)**		x		x
**PaO_2/FiO^2**				
**SGPT**		x		x
**SGOT**		x		x
**S.Ferritin**		x		x
**CXR**		x	x	

**6.Treatment**	
Oxygen therapy	Yes/No Total duration (in hours):
IV Fluid	Yes/No, How much (in litres):
Pressure support required	Yes/No, How long (in hours):
Mechanical Ventilation required	Yes/No, if yes duration (days):
Dialysis required	Yes/No, How many cycles:
Steroid	Yes/No
Any antiviral treatment Yes/No	Mention name:<br>HCQ: Yes/No<br>AZYTH: Yes/No
Antibiotic	Yes/No, if yes duration (days)
Enoxaparin	Yes/No, if yes duration (days)

**7. Duration of ICU stay (in days)**	
**8. Duration of hospital stay (in days)**	

9. Outcome (Tick) **10. Date of Discharge:**

Recovered	Died

Signature of the concerned physician

Annex II: PIS and Consent for Donor

গবেষণা সংক্রান্ত তথ্যাবলি ও সম্মতি পত্র

কোভিড-১৯ থেকে সুস্থ হওয়া রুগীদের জন্য

অধ্যয়নের বিষয়বস্তু: বাংলাদেশের যেসব মানুষের করোনায় আক্রান্ত হয়েছেন তাদের ক্ষেত্রে সুস্থ হয়ে যাওয়া মানুষের রক্ত থেকে পৃথক করা উপাদান(প্লাজমা) ব্যবহারের উপকারিতা রয়েছে কিনা তা পর্যবেক্ষণ করা।

গবেষণার বিষয়বস্তু কি?

কোভিড-১৯ এ আক্রান্ত হয়ে সমগ্র পৃথিবীতে মৃত্যুর হার ব্যাপক। এটি একটি সংক্রামক ব্যাধি। বাংলাদেশেও এই রোগের প্রাদুর্ভাব দেখা দিয়েছে। নানা উপসর্গ সম্বলিত এই রোগের অনেক কিছুই সাধারন অন্য রোগের মত। একেবারে সুনির্দিষ্ট কোনও ওষুধ এখন পর্যন্ত প্রতিষ্ঠিত না হওয়াতে অনেক রুগীর চিকিৎসা অপূর্ণ থেকে যায়। ফলে মৃত্যুর ঝুঁকি থেকেই যায়। যেসকল রুগী সুস্থতা লাভ করে তাদের শরীরে রোগটির বিরুদ্ধে প্রতিরোধ ক্ষমতা তৈরি হয় এবং এটি রুগী ভেদে তারতম্য থাকে কার ভিতরে কতটুকু ক্ষমতা অর্জন হয়েছে। সেই ক্ষমতা বৈজ্ঞানিক ভাবে নিরূপণ করে অন্য সুস্থ মানুষের থেকে উপাদানটি(প্লাজমা) পৃথক করে তাকে অন্য রুগীর সুস্থতা অর্জনে ব্যবহার করা যায়। সাম্প্রতিক সময়ে বিশ্বব্যাপী এইভাবে গুরুতর অসুস্থ রুগীদের ক্ষেত্রে এটি প্রয়োগ করে বেশ কিছু সফলতা অর্জন করা সম্ভব হয়েছে।

এই গবেষণায় আমরা কোভিড-১৯ থেকে সুস্থ হয়ে যাওয়া রুগীদের রক্তে উপস্থিত প্রতিষেধকের মাত্রা দেখবো এবং এটি অন্য গুরুতর রুগীর ক্ষেত্রে এটি কিভাবে ব্যবহার করা যায় তা অনুসন্ধান করবো।

কেন আমাকে ডাকা হয়েছে:

আপনি এই হাসপাতালে কোভিড-১৯ নিয়ে ভর্তি হয়েছেন / অন্যত্র অবস্থান করছেন। এই অধ্যয়নে আমরা আপনার শরীরে কোভিড-১৯ এর এন্টিবডির মাত্রা অনুসন্ধান এবং প্রয়োজনে একে অন্যজনের ক্ষেত্রে প্রয়োগ করা যায় কিনা তার সম্ভবতা অনুসন্ধান করতে চাই।

আমাকে কি করতে হবে ?

আপনি যদি অংশগ্রহণ করতে চান তাহলে আপনাকে রক্ত দিতে বলা হবে (১০ মিলিলিটার বা ২ টেবিল চামিচের মতো) । কোভিড-১৯ এর এন্টিবডি নির্ণয়ের কাজে তা ব্যবহার করা হবে। অন্য গুরুতর রুগীর ক্ষেত্রে প্রয়োগ করার মত কার্যক্ষম এন্টিবডি যদি আপনার রক্তে পাওয়া যায় তবে সেক্ষেত্রে স্বাভাবিক রক্তদানের যেসকল পরীক্ষা করা হয় তা সুনিপুণ ভাবে করার পর এফেরেসিস মেশনের মাধ্যমে ৫০০ মিলি প্লাজমা আপনার শরীর থেকে সংগ্রহ করা হবে। এতে আপনার স্বাস্থ্যের কোনও রকম ঝুঁকি না হওয়ার সম্ভাবনাই সব থেকে বেশী। যে সকল ঝুঁকি হওয়ার সম্ভাবনা থাকে তা জীবন সংহারী নয়। রক্ত সংগ্রহের জন্য কাজ করবেন একজন ডাক্তার বা একজন প্রশিক্ষন প্রাপ্ত সিনিয়র সেবিকা। আমরা আপনার কিছু সাধারণ ব্যক্তিগত তথ্য এবং আপনার অসুস্থ্যতার ইতিহাস চাইব। আপনার বাসার ঠিকানা এবং টেলিফোন নাম্বার আমাদেরকে দিতে হবে। আপনি কেমন আছেন সেটা জানার জন্য আমরা ২৮ দিন পর আপনার সাথে যোগাযোগ করব।

সম্ভাব্য সময়কাল:

আমাদের গবেষণায় অংশগ্রহণের জন্য প্রথমবার মোটামুটি ৪০ মিনিট সময় লাগবে। রক্তে কোভিড-১৯ প্রতিরোধী এন্টিবডি া পাওয়া গেলে পরবর্তী পরীক্ষার রক্ত সংগ্রহের জন্য আরও ৪০ মিনিট সময় প্রয়োজন হতে পারে।

আমার তথ্য/নমুনার কি হবে:

আপনার ব্যক্তিগত এবং অসুস্থ্যতা সর্ম্পকিত তথ্য একটা ফরমে লিপিবদ্ধ থাকবে। সেখানে আপনার বিস্তারিত ব্যক্তিগত বিষয় থাকবে না যেটা আপনাকে সনাক্ত করতে পারে। পাসওয়ার্ড সংরক্ষিত ডাটাবেজে এই তথ্য সংরক্ষিত হবে। তথ্যের গোপনীয়তা পূর্ণভাবে বজায় থাকবে। আমাদের গবেষণা সহকারী হবে ডাক্তার যাদেরকে তথ্য সংগ্রহ এবং নথিভুক্ত করার

জন্য নিয়োগ দেওয়া হবে। গবেষণা শেষ করার পর এই তথ্য কাগজে থাকবে কমপক্ষে ৩ বছর এবং কম্পিউটারে থাকবে কমপক্ষে ৫ বছর। শুধুমাত্র প্রধান এবং সহকারী গবেষকদেরকে এই তথ্য দেখার অনুমতি দেওয়া হবে।

আপনার ব্যক্তিগত তথ্য অন্যের কাছে প্রকাশ করা হবে না। যখন গবেষণা শেষ হবে আমরা অন্যান্য অংশগ্রহণকারীর সাথে আপনার ফলাফলটা একত্রিত করব এবং সমস্ত ফলাফল বিশ্লেষণ করা করব। ফলফল সমূহ একটি বৈজ্ঞানিক গবেষণা আকারে প্রকাশ করা হবে। যেখানে আমরা নিশ্চয়তা দিতে পারি যে, আপনার ব্যক্তিগত তথ্য প্রকাশ করা হবে না। শুধুমাত্র অজানা একটি দলের রোগীদের মোট ফলাফল প্রকাশ করা হবে। এই গবেষণার বাইরে যদি আমরা অন্য কোন নতুন গবেষণা করতে চাই তাহলে নীতি নির্ধারণী কমিটির কাছে সেটার জন্য আবেদন করা হবে।

<u>অংশগ্রহণ করার কোন ঝুঁকি আছে কি:</u>

এই গবেষণায় কোন শারিরীক বা মনস্তাত্বিক ঝুঁকি নেই। নমুনা সংগ্রহের ক্ষেত্রে একটু ব্যথা, একটু রক্ত বের হবে যার জন্য সাধারণ কোন চিকিৎসা করা লাগবে না। প্লাজমা সংগ্রহের সময় শরীরে হাল্কা শিতশিত অনুভুত হওয়া, হাল্কা থেকে মাঝারি মাথা ব্যাথা, বমি লাগা ইত্যাদি লাগতে পারে যা আমাদের চিকিৎসক দল সঠিক চিকিৎসা দিয়ে দিবেন। কোনও প্রকার আর্থিক ক্ষতিপূরণ করা হবেনা।

<u>অংশ গ্রহণ করার সুবিধা কি:</u>

আপনি বিনামূল্যে আপনার রক্তের মুদ্রিত ফলাফল পাবেন (কোভিড-১৯ এন্টিবডি)। এছাড়া ও বিনামূল্যে আপনার হেপাটাইটিস বি ও সি ভাইরাস, এইচআইভি ভাইরাস, সিফিলিস, পরীক্ষা করা হবে এবং কাগজে একটি কপি আপনাকে দেওয়া হবে । এছাড়া আপনি অন্য কোন সরাসরি সুবিধা এই গবেষণা থেকে পাবেন না। এই গবেষণার ফলাফল খুবই গুরুত্বপূর্ণ।

<u>আমাকে কি অংশগ্রহণ করতেই হবে:</u>

না। গবেষণায় অংশগ্রহণ স্বেচ্ছা প্রদত্ত। কোন কারণ ছাড়া গবেষণার যেকোন পর্যায়ে আপনি আপনার মন পরিবর্তন করতে পারবেন। যদি আপনি গবেষণা থেকে সরে দাড়ান তাহলে সরে দাড়ানো আগে সংরক্ষিত রক্ত ব্যবহার করা হবে যদি না আপনি বিশেষভাবে অন্য কিছুর জন্য অনুরোধ করেন। যদি আপনি গবেষণায় রাজী না হন তাহলেও হাসপাতালের অন্যান্য রোগীদের মতো একই চিকিৎসা পাবেন। এটা আপনার চিকিৎসার উপর কোন নেতিবাচক প্রভাব ফেলবে না। আপনি যেকোন সময় প্রশ্ন করতে পারবেন।

তারিখ:

আমি--------------------------------(নাম), আমাকে গবেষণার বিষয়বস্তু বিষদ ভাবে ব্যাখ্যা করা হয়েছে।
গবেষণা সহকারী .... আমার সাথে আলোচনা করেছেন।

আমাকে নিচের বিষয়গুলো অবহিত করেছেন

-গবেষণার উদ্দেশ্য এবং গবেষণায় অংশগ্রহণের জন্য প্রয়োজনীয় সময়।
-যে পদ্ধতির মাধ্যমে অনুষ্ঠিত হবে।
-গবেষণার প্রত্যাশিত ফলাফল।
-গবেষণায় অংশগ্রহণের ঝুঁকি।

পড়া হয়েছে আমি সব বুঝেছি এবং সন্তোষজনক ভাবে সব প্রশ্নের উত্তর পেয়েছি।

নিচের প্রয়োজনীয় ঘরগুলো পূরণ করুন:

- ➢ আমি গবেষণায় অংশগ্রহণ করতে প্রস্তুত/প্রস্তুত না।
- ➢ আমি আমার অবশিষ্ট রক্ত এবং অন্যান্য নমুনা সংরক্ষণ করতে এবং ৩ বছর ভবিষ্যৎ গবেষণারজন্য প্রদান করতে অনুমতি প্রদান করি/অনুমতি প্রদান করিনা।
- ➢ আমি যদি এই অধ্যয়ন থেকে সরে দাড়াই তাহলে আমার রক্ত সংরক্ষণ করতে বা পওে ব্যবহার করতে অনুমতি প্রদান করি/অনুমতি প্রদান করিনা।

➢ বাংলাদেশের বাইরে কোন গবেষণা প্রতিষ্ঠানের জন্য আমি রক্ত প্রদান করতে অনুমতি প্রদান করি/অনুমতি প্রদান করিনা।

আমি বুঝতে পারছি যে, আমি/অংশগ্রহণকারী যেকোন সময় মতামত পরিবর্তন করতে পারি। এই গবেষণায় অংশগ্রহণের সম্মতিতে আমি গবেষকদেও আমার ব্যক্তিগত তথ্য ব্যবহার করার অনুমতি দেই। গবেষণার অংশ হিসেবে কখনোই আমার নাম প্রকাশ করা হবেনা।

অংশগ্রহণকারীর স্বাক্ষর-----------------------------তারিখ--------------------------

অংশগ্রহণকারীর নাম-----------------------------------সময়-----------------------

অথবা যদি অংশগ্রহণকারী অনুমতি দিতে সামর্থ্য না থাকে

অংশগ্রহণকারী প্রতিনিধির স্বাক্ষর---------------------------------তারিখ------------------

অংশগ্রহণকারী প্রতিনিধির নাম-----------------------------------সময়------------------

যদি অংশগ্রহণকারী পড়তে বা লিখতে না পারেন তাহলে প্রয়োজনীয় তথ্যঃ
গবেষণাসহকারী যাবতীয় তথ্য আমার সামনে পড়েছেন এবংবর্ণনা করেছেন। আমি প্রদেয় তথ্য সম্পূর্ণভাবে বুঝতে পেরেছি। তাই আমি এই গবেষণায় অংশগ্রহণের সম্মতির জন্য হস্ত স্বাক্ষর দিয়েছি।

স্বাক্ষীর স্বাক্ষর------------------------
স্বাক্ষীর নাম--------------------------
তারিখ------------------------------
সময়------------------------------

অংশগ্রহণকারীর ডান হাতের
বৃদ্ধা আঙ্গুলির ছাপ

স্বাক্ষী এমন একজন ব্যক্তি যে এই গবেষণার বাইরের কেউ
বা এই দলের একজন সদস্য যে অনুমতি অর্জনে অন্তর্ভূক্ত ছিলেন না

গবেষকপূরণ করবেনঃ
আমি প্রত্যয়ন করছি যে, অংশগ্রহণকারীদেও কাছ থেকে অনুমতি নেওয়ার জন্য আমি সমস্ত নিয়মাবলী অনুসরণ করেছি।
তিনি এই গবেষণার উদ্দেশ্য পরিস্কার ভাবে বুঝেছেন এবং গবেষণায় অংশগ্রহণের জন্য অনুমতি দিয়েছেন। তাকে প্রশ্ন করার সুযোগ দেওয়া হয়েছে এবং প্রশ্নের উত্তর সন্তোষজনকভাবে দেওয়া হয়েছে।

গবেষকের স্বাক্ষর-----------------------------তারিখ----------------

গবেষকের নাম -------------------------------সময়-----------------

স্বাক্ষরিত একটি ফটোকপি অংশগ্রহণকারীকে সংরক্ষণের জন্য দেওয়া হবে।

Annex III: PIS and Consent for Patient

গবেষণা সংক্রান্ত তথ্যাবলি ও সম্মতি পত্র

কোভিড-১৯ আক্রান্ত রুগীর জন্য

অধ্যয়নের বিষয়বস্তু: বাংলাদেশের যেসব মানুষের করোনায় আক্রান্ত হয়েছেন তাদের ক্ষেত্রে সুস্থ হয়ে যাওয়া মানুষের রক্ত থেকে পৃথক করা উপাদান(প্লাজমা) আক্রান্ত রুগীদের ক্ষেত্রে প্রয়োগের ফলে সেসকল উপকারিতা পাওয়া সম্ভব তা বৈজ্ঞানিক ভাবে পর্যবেক্ষণ করা।

গবেষণার বিষয়বস্তু কি?

কোভিড-১৯ এ আক্রান্ত হয়ে সমগ্র পৃথিবীতে মৃত্যুর হার ব্যাপক। এটি একটি সংক্রামক ব্যাধি। বাংলাদেশেও এই রোগের প্রাদুর্ভাব দেখা দিয়েছে। নানা উপসর্গ সম্বলিত এই রোগের অনেক কিছুই সাধারন অন্য রোগের মত। একেবারে সুনির্দিষ্ট কোনও ওষুধ এখন পর্যন্ত প্রতিষ্ঠিত না হওয়াতে অনেক রুগীর চিকিৎসা অপূর্ণ থেকে যায়। ফলে মৃত্যুর ঝুঁকি থেকেই যায়। যেসকল রুগী সুস্থতা লাভ করে তাদের শরীরে রোগটির বিরুদ্ধে প্রতিরোধ ক্ষমতা তৈরি হয় এবং এটি রুগী ভেদে তারতম্য থাকে কার ভিতরে কতটুকু ক্ষমতা অর্জন হয়েছে। সেই ক্ষমতা বৈজ্ঞানিক ভাবে নিরূপণ করে অন্য সুস্থ মানুষের থেকে উপাদানটি পৃথক করে তাকে অন্য রুগীর সুস্থতা অর্জনে ব্যবহার করা যায়। সাম্প্রতিক সময়ে বিশ্বব্যাপী এইভাবে গুরুতর অসুস্থ রুগীদের ক্ষেত্রে এটি প্রয়োগ করে বেশ কিছু সফলতা অর্জন করা সম্ভব হয়েছে।

এই গবেষণায় আমরা কোভিড-১৯ থেকে সুস্থ হয়ে যাওয়া রুগীদের রক্তে উপস্থিত প্রতিষেধকের মাত্রা দেখে রোগাক্রান্ত রুগীদের শরীরে প্রবেশ করিয়ে তার ধারাবাহিক ফলাফল অনুসন্ধান করবো ।

কেন আমাকে ডাকা হয়েছে:

আপনি এই হাসপাতালে কোভিড-১৯ নিয়ে ভর্তি হয়েছেন । এই অধ্যয়নে আমরা সুস্থ মানুষের শরীর থেকে পর্যাপ্ত ও পরীক্ষিত প্লাজমা সংগ্রহ করে তা আপনার শরীরে প্রদান করা হবে। পরবর্তীতে কয়েকধাপে পরীক্ষা করে দেখা হবে পরিবর্তন সমুহ।

আমাকে কি করতে হবে

আপনি যদি অংশগ্রহণ করতে চান তবে পর্যাপ্ত পরীক্ষা নিরীক্ষা করার পর ২০০/400 মিলিলিটার প্লাজমা যাতে পর্যাপ্ত পরিমান কোভিড-১৯ এর বিপক্ষে কার্যকরী এন্টিবডি রয়েছে তা আপনার রক্তে প্রবেশ করানো হবে। এরপর ধাপে ধাপে আপনার রক্তের ও শরীরের নানাবিধ পরিবর্তন পর্যবেক্ষণ করা হবে।

এতে আপনার স্বাস্থ্যের কোনও রকম ঝুঁকি না হওয়ার সম্ভাবনাই সব থেকে বেশী। যে সকল ঝুঁকি হওয়ার সম্ভাবনা থাকে তা জীবন সংহারী নয়।রক্ত প্রদানকালীন একজন চিকিৎসক আপনার পাশে অবস্থান করবেন। আমরা আপনার কিছু সাধারণ ব্যক্তিগত তথ্য এবং আপনার অসুস্থ্যতার ইতিহাস চাইব । আপনার বাসার ঠিকানা এবং টেলিফোন নাম্বার আমাদেরকে দিতে হবে । আপনি কেমন আছেন সেটা জানার জন্য আমরা ২৮ দিন পর আপনার সাথে যোগাযোগ করব ।

সম্ভাব্য সময়কাল:

আমাদের গবেষণায় অংশগ্রহণের জন্য প্রথমবার মোটামুটি ১২০ মিনিট সময় লাগবে। রক্তে কোভিড-১৯ প্রতিরোধী এন্টিবডি৷ পাওয়া গেলে পরবর্তী পরীক্ষার রক্ত সংগ্রহের জন্য আরও ৪০ মিনিট সময় প্রয়োজন হতে পারে।

আমার তথ্য/নমুনার কি হবে:

আপনার ব্যক্তিগত এবং অসুস্থ্যতা সর্ম্পকিত তথ্য একটা ফরমে লিপিবদ্ধ থাকবে। সেখানে আপনার বিস্তারিত ব্যক্তিগত বিষয় থাকবে না যেটা আপনাকে সনাক্ত করতে পারে। পাসওয়ার্ড সংরক্ষিত ডাটাবেজে এই তথ্য সংরক্ষিত হবে। তথ্যের গোপনীয়তা পূর্ণভাবে বজায় থাকবে। আমাদের গবেষণা সহকারী হবে ডাক্তার যাদেরকে তথ্য সংগ্রহ এবং নথিভুক্ত করার জন্য নিয়োগ দেওয়া হবে। গবেষণা শেষ করার পর এই তথ্য কাগজে থাকবে কমপক্ষে ৩ বছর এবং কম্পিউটারে থাকবে কমপক্ষে ৫ বছর। শুধুমাত্র প্রধান এবং সহকারী গবেষকদেরকে এই তথ্য দেখার অনুমতি দেওয়া হবে।

আপনার ব্যক্তিগত তথ্য অন্যের কাছে প্রকাশ করা হবে না। যখন গবেষণা শেষ হবে আমরা অন্যান্য অংশগ্রহণকারীর সাথে আপনার ফলাফলটা একত্রিত করব এবং সমস্ত ফলাফল বিশ্লেষণ করা করব। ফলফল সমূহ একটি বৈজ্ঞানিক গবেষণা আকারে প্রকাশ করা হবে। যেখানে আমরা নিশ্চয়তা দিতে পারি যে, আপনার ব্যক্তিগত তথ্য প্রকাশ করা হবে না। শুধুমাত্র অজানা একটি দলের রোগীদের মোট ফলাফল প্রকাশ করা হবে। এই গবেষণার বাইরে যদি আমরা অন্য কোন নতুন গবেষণা করতে চাই তাহলে নীতি নির্ধারণী কমিটির কাছে সেটার জন্য আবেদন করা হবে।

অংশগ্রহণ করার কোন ঝুঁকি আছে কি:

এই গবেষণায় কোন শারিরীক বা মনস্তাত্বিক ঝুঁকি নেই। সাধারণভাবে রক্ত পরিসঞ্চালন কালীন যেসকল সমস্যা যেমন হাল্কা চুলকানি, মাথা ধরা, বমি ভাব হওয়া ইত্যাদি হতে পারে। যা আমাদের চিকিৎসক দল সঠিক চিকিৎসা দিয়ে দিবেন। কোনও প্রকার আর্থিক ক্ষতিপূরণ করা হবেনা।

আমাকে কি অংশগ্রহণ করতেই হবে:

না। গবেষণায় অংশগ্রহণ স্বেচ্ছা প্রদত্ত। কোন কারণ ছাড়া গবেষণার যেকোন পর্যায়ে আপনি আপনার মন পরিবর্তন করতে পারবেন। যদি আপনি গবেষণা থেকে সরে দাড়ান তাহলে সরে দাড়ানো আগে সংরক্ষিত রক্ত ব্যবহার করা হবে যদি না আপনি বিশেষভাবে অন্য কিছুর জন্য অনুরোধ করেন। যদি আপনি গবেষণায় রাজী না হন তাহলেও হাসপাতালের অন্যান্য রোগীদের মতো একই চিকিৎসা পাবেন। এটা আপনার চিকিৎসার উপর কোন নেতিবাচক প্রভাব ফেলবে না। আপনি যেকোন সময় প্রশ্ন করতে পারবেন।

তারিখ:

আমি-------------------------------(নাম), আমাকে গবেষণার বিষয়বস্তু বিষদ ভাবে ব্যাখ্যা করা হয়েছে।
গবেষণা সহকারী, আমার সাথে আলোচনা করেছেন।

আমাকে নিচের বিষয়গুলো অবহিত করেছেন

-গবেষণার উদ্দেশ্য এবং গবেষণায় অংশগ্রহণের জন্য প্রয়োজনীয় সময়।

-যে পদ্ধতির মাধ্যমে অনুষ্ঠিত হবে।

-গবেষণার প্রত্যাশিত ফলাফল।

-গবেষণায় অংশগ্রহণের ঝুঁকি।

পড়া হয়েছে আমি সব বুঝেছি এবং সন্তোষজনক ভাবে সব প্রশ্নের উত্তর পেয়েছি।

নিচের প্রয়োজনীয় ঘরগুলো পূরণ করুন:

- আমি গবেষণায় অংশগ্রহণ করতে প্রস্তুত/প্রস্তুত না।
- আমি আমার অবশিষ্ট রক্ত এবং অন্যান্য নমুনা সংরক্ষণ করতেএবং ৩ বছর ভবিষ্যৎ গবেষণার জন্য প্রদান করতে অনুমতি প্রদান করি/অনুমতি প্রদান করিনা।
- আমি যদি এই অধ্যয়ন থেকে সরে দাড়াই তাহলে আমার রক্ত সংরক্ষণ করতে বা পওে ব্যবহার করতে অনুমতি প্রদান করি/অনুমতি প্রদান করিনা।
- বাংলাদেশের বাইরে কোন গবেষণা প্রতিষ্ঠানের জন্য আমি রক্ত প্রদান করতে অনুমতি প্রদান করি/অনুমতি প্রদান করিনা।

আমি বুঝতে পারছি যে, আমি/অংশগ্রহণকারী যেকোন সময় মতামত পরিবর্তন করতে পারি। এই গবেষণায় অংশগ্রহণের সম্মতিতে আমি গবেষকদেও আমার ব্যক্তিগত তথ্য ব্যবহার করার অনুমতি দেই। গবেষণার অংশ হিসেবে কখনোই আমার নাম প্রকাশ করা হবেনা।

অংশগ্রহণকারীর স্বাক্ষর-----------------------------তারিখ--------------------------

অংশগ্রহণকারীর নাম-----------------------------------সময়-----------------------

অথবা যদি অংশগ্রহণকারী অনুমতি দিতে সামর্থ্য না থাকে

অংশগ্রহণকারী প্রতিনিধির স্বাক্ষর--------------------------------তারিখ------------------

অংশগ্রহণকারী প্রতিনিধির নাম-----------------------------------সময়------------------

যদি অংশগ্রহণকারী পড়তে বা লিখতেনা পারেন তাহলে প্রয়োজনীয় তথ্য:

গবেষণা সহকারী যাবতীয় তথ্য আমার সামনে পড়েছেন এবং বর্ণনা করেছেন। আমি প্রদেয় তথ্য সম্পূর্ণভাবে বুঝতে পেরেছি। তাই আমি এই গবেষণায় অংশগ্রহণের সম্মতির জন্য হস্তস্বাক্ষর দিয়েছি।

স্বাক্ষীর স্বাক্ষর------------------------

স্বাক্ষীরনাম--------------------------

তারিখ------------------------------

সময়------------------------------

অংশগ্রহণকারীর ডান হাতের
বৃদ্ধা আঙ্গুলির ছাপ

স্বাক্ষীএমনএকজন ব্যক্তি যে এই গবেষণারবাইরের কেউ
বা এই দলের একজন সদস্য যে অনুমতি অর্জনে অন্তর্ভূক্ত ছিলেন না

গবেষকপূরণ করবেন:

আমি প্রত্যয়ন করছি যে, অংশগ্রহণকারীদেও কাছ থেকে অনুমতি নেওয়ার জন্য আমি সমস্ত নিয়মাবলী অনুসরণ করেছি।

তিনি এই গবেষণার উদ্দেশ্য পরিস্কারভাবে বুঝেছেন এবং গবেষণায় অংশগ্রহণের জন্য অনুমতি দিয়েছেন। তাকে প্রশ্ন করার সুযোগ দেওয়া হয়েছে এবং প্রশ্নের উত্তর সন্তোষজনকভাবে দেওয়া হয়েছে।

গবেষকের স্বাক্ষর-----------------------------তারিখ----------------

গবেষকেরনাম --------------------------------সময়-----------------

স্বাক্ষরিত একটি ফটোকপি অংশগ্রহণকারীকে সংরক্ষণের জন্য দেওয়াহবে।

Annex-IV: Dummy table

	Standard (n=20)	CP 200 (n=20)	CP 400 (n= 20)			
Clinical						
Time required to gain normal saturation (hours; median, IQR)				p= (by Kruskal-Wallis)		
Duration of hospital stay (days; median, IQR)				p= (by Kruskal-Wallis)		
Amount of oxygen required (litres; median, IQR)				p= (by Kruskal-Wallis)		
					**Odds ratios**	
Laboratory				**CP200** vs **Standard**	**CP400** vs **Standard**	**CP200** vs **CP400**
Neutrophil/Lymphocyte ratio (in %)						
Lymphocyte count (number)						
CRP (mg/L)						
Ferritin (ng/ml)						
SGPT (IU/L)						
Outcome						
Death						
Discharged without complication						
